# Kimura Disease Presenting as Cervical Lymphadenopathy with Marked Eosinophilia in a Saudi Adolescent: A Case Report and Literature Review

**DOI:** 10.3390/healthcare14131956

**Published:** 2026-07-02

**Authors:** Shaima Al Aoun, Khaled Abdulwahab Amer, Raghad Saeed Asiri, Houda Gharsalli, Shimaa Saad Elkholy

**Affiliations:** 1 Division of Hematology, Aseer Central Hospital, Aseer Health Cluster, Abha 62523, Saudi Arabia; salaoun@kku.edu.sa; 2 Department of Internal Medicine, Aseer Central Hospital, Aseer Health Cluster, Abha 62523, Saudi Arabia; 2ragahd2@gmail.com; 3 Division of Pulmonology, Aseer Central Hospital, Aseer Health Cluster, Abha 62523, Saudi Arabia; drhoudagharsalli@gmail.com; 4 Department of Anatomic Pathology, Aseer Central Hospital, Aseer Health Cluster, Abha 62523, Saudi Arabia; shimaaelkholy2020md@gmail.com

**Keywords:** Kimura disease, eosinophilia, cervical lymphadenopathy, immunoglobulin E, lymphadenopathy, pediatric, Saudi Arabia, case report

## Abstract

**Background/Objectives**: Kimura disease is a rare, chronic immune-mediated disorder that classically produces painless head-and-neck masses, regional lymphadenopathy, blood eosinophilia, and a raised serum immunoglobulin E (IgE). Almost all reported patients are young East Asian men, and the condition is seldom encountered in the Middle East, where it is easily mistaken for lymphoma or another eosinophilic disorder. We describe a histologically confirmed case in a Saudi adolescent and review the literature to compare treatment strategies and outcomes across populations. **Methods**: We documented the clinical course, laboratory profile, histopathological findings, and 15-month outcome of the patient, and searched PubMed for reported cases of Kimura disease, with emphasis on pediatric and non-Asian series. **Results**: A 16-year-old boy presented with a one-year history of painless right cervical swelling, constitutional symptoms and striking eosinophilia (36%; absolute eosinophil count 6.5 × 10^9^/L). Hematological malignancy was excluded through bone-marrow examination, flow cytometry and molecular studies. Excisional lymph node biopsy revealed the diagnostic triad—reactive follicular hyperplasia, a dense eosinophilic infiltrate with microabscesses, and vascular proliferation—together with IgE-positive immunostaining. Complete remission followed surgical excision alone and was maintained at 15 months without systemic corticosteroids. **Conclusions**: Kimura disease occurs well beyond its traditional geographic boundaries and belongs in the differential diagnosis of eosinophilia accompanied by lymphadenopathy. Although corticosteroids remain the mainstay for extensive disease, relapse on tapering is common, whereas complete excision can secure lasting remission in localized pediatric disease while sparing patients from steroid-related toxicity.

## 1. Introduction

First documented in the Chinese literature by Kimm and Szeto in 1937 under the name “eosinophilic hyperplastic lymphogranuloma” and characterized more fully a decade later by Kimura and colleagues, who drew attention to its peculiar vascular and connective-tissue changes, Kimura disease (KD) is an uncommon chronic inflammatory condition of uncertain cause [1,2]. It is recognized clinically by a triad of slowly enlarging, painless subcutaneous masses with a predilection for the head and neck, accompanying regional lymph node enlargement, and a hematological signature of peripheral eosinophilia with an elevated serum immunoglobulin E (IgE) [3,4].

The disorder shows pronounced demographic clustering. Most large series originate from China, Japan and Korea, and young men are affected far more often than women, with reported male-to-female ratios between roughly 3.5:1 and 9:1 and a usual onset in the third and fourth decades of life [5,6,7]. Pediatric presentations, once regarded as exceptional, are increasingly described: Chinese cohorts report a median age at diagnosis in the early teens and an even steeper male predominance [8,9].

Outside East Asia, the disease is genuinely scarce, and clinicians unfamiliar with it frequently embark on an extensive malignancy workup before the correct diagnosis emerges [4,10]. Reports from the Arabian Peninsula are particularly sparse; to our knowledge, only a single pediatric Saudi case has previously been published [11]. This unfamiliarity matters because the combination of a neck mass, eosinophilia, and constitutional symptoms readily raises concern for lymphoma or a primary eosinophilic neoplasm, and the diagnostic emphasis shifts accordingly.

The pathogenesis of KD remains incompletely defined, but the prevailing view implicates a skewed T-helper-2 (Th2) immune response. Supporting evidence includes raised tissue and circulating concentrations of interleukin-4 (IL-4), IL-5 and IL-13 and of tumor necrosis factor-alpha (TNF-α), which together drive eosinophil recruitment and IgE class-switching [12,13]. Consistent with this mechanism, the disease is frequently accompanied by atopic conditions such as asthma, allergic rhinitis and eczema [14].

There is no universally agreed standard of care for KD, reflecting both its rarity and the absence of randomized trials. Management is therefore individualized and spans active surveillance after complete excision, systemic corticosteroids, radiotherapy, and steroid-sparing or biologic agents. In practice, complete surgical excision is generally favored for localized, resectable lesions, whereas corticosteroids are most often used first-line for extensive, multifocal, or recurrent disease, accepting that relapse during tapering is frequent [13,15].

We present what is, to our knowledge, the second pediatric case of KD reported from Saudi Arabia and one of very few from the wider region. The case is distinguished by durable remission achieved with surgical excision alone, without systemic corticosteroids. We pair the report with a focused review of the literature that compares treatment approaches and outcomes across geographic and age groups.

## 2. Case Presentation

A 16-year-old Saudi boy of Arab ethnicity with well-controlled bronchial asthma maintained on a low-dose inhaled corticosteroid was referred in February 2024 to the Hematology Clinic at Aseer Central Hospital, Aseer Health Cluster, Abha, Saudi Arabia, for a painless right-sided neck swelling that had been present for roughly one year. Over the preceding six months, he had unintentionally lost about 5 kg, experienced drenching night sweats several times each week, and noted intermittent low-grade fever. He reported no rash, pruritus, joint pain, or features suggestive of active infection. His only relevant surgical history was the childhood excision of a benign bone tumor (histologically an osteochondroma of the distal right femur, removed at approximately 8 years of age).

On examination, he was well-nourished and in no distress. Several firm, non-tender, mobile nodes were palpable along the right cervical chain, the largest measuring approximately 2 × 2 cm. There were no overlying skin changes, subcutaneous nodules or salivary-gland enlargement, and no hepatosplenomegaly, generalized lymphadenopathy or peripheral edema. Vital signs were unremarkable.

Initial blood work showed marked leukocytosis, with a white-cell count of 40 × 10^9^/L (reference 4.5–11.0 × 10^9^/L). The differential was dominated by eosinophils, which accounted for 36% of leukocytes and an absolute eosinophil count of 6.5 × 10^9^/L. Hemoglobin and platelet counts were normal. A peripheral smear confirmed prominent, morphologically mature eosinophilia without blasts, atypical cells or dysplasia.

To exclude a hematological malignancy, bone-marrow aspiration and trephine biopsy were undertaken. These showed a hypercellular marrow with reactive, hyperplastic granulopoiesis and abundant eosinophils, but no evidence of leukemia, lymphomatous infiltration, or myelodysplasia. Flow cytometry detected no clonal B- or T-cell population. Molecular testing was negative for the *BCR-ABL1* fusion, *JAK2* V617F, *CALR* and *MPL* mutations, and fluorescence in situ hybridization showed no *PDGFRB* rearrangement, effectively ruling out chronic myeloid leukemia and myeloid/lymphoid neoplasms with eosinophilia (Table 1).

An autoimmune and vasculitis screen was unremarkable: both cytoplasmic and perinuclear antineutrophil cytoplasmic antibodies (c-ANCA and p-ANCA) were negative. Serum tryptase was normal, arguing against systemic mastocytosis, while serum IgE was markedly elevated at 2480 IU/mL (reference range < 100 IU/mL). Stool examination for ova and parasites, serology for Strongyloides and Toxocara, and a fourth-generation HIV assay were all negative, excluding common secondary causes of marked eosinophilia. Contrast-enhanced computed tomography of the neck, chest, abdomen and pelvis confirmed isolated right cervical lymphadenopathy with no other nodal involvement, in keeping with localized disease (Table 1).

**Figure 1:**
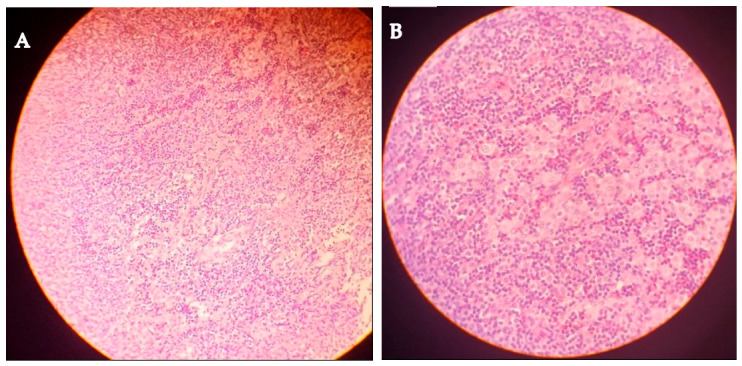
Excisional biopsy of the right cervical lymph node (hematoxylin and eosin). (**A**) Low-power view showing reactive follicular hyperplasia with preserved nodal architecture; (**B**) high-power view showing a dense interfollicular infiltrate of mature eosinophils.

**Figure 2:**
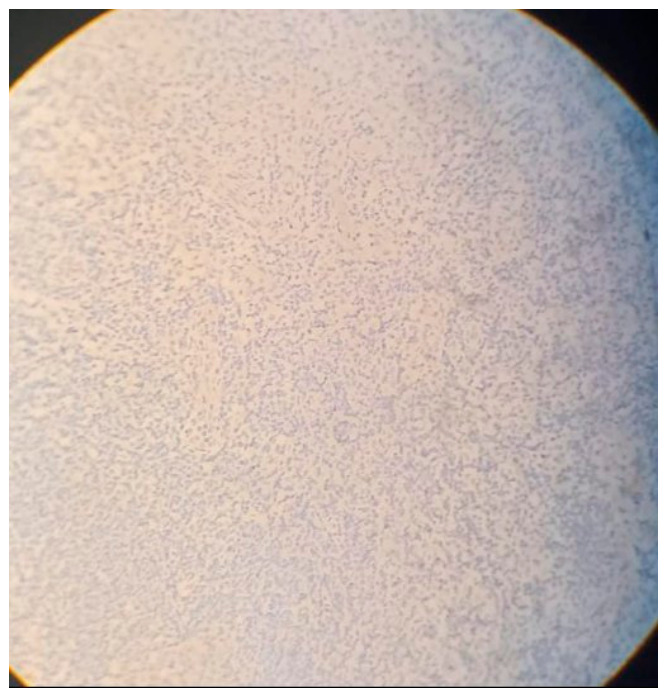
Immunohistochemistry showing negative CD1a staining, which argues against Langerhans cell histiocytosis.

**Figure 3:**
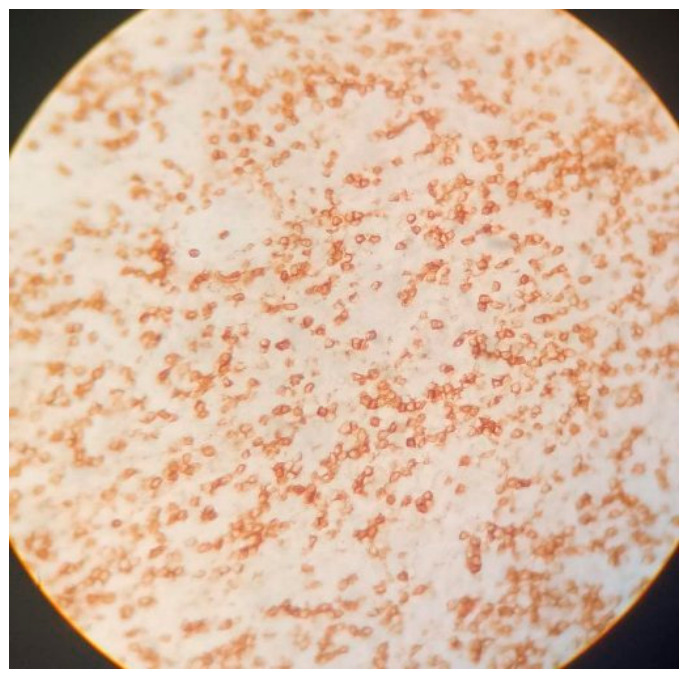
Immunohistochemistry for IgE demonstrating extensive interfollicular positivity, a feature characteristic of Kimura disease.

An excisional biopsy of the largest right cervical node was performed for definitive diagnosis. Histology displayed the characteristic triad of KD: reactive follicular hyperplasia with preserved nodal architecture; a dense interfollicular eosinophilic infiltrate with eosinophilic microabscess formation; and proliferation of post-capillary venules (Figure 1). There were no Reed–Sternberg cells, atypical lymphoid populations, or granulomatous inflammation. On immunohistochemistry, CD1a and Langerin were negative, excluding Langerhans cell histiocytosis (Figure 2), whereas IgE staining highlighted extensive interfollicular positivity—a finding strongly supportive of KD (Figure 3).

Because the disease was localized and the diagnostic excision had already removed the dominant nodal mass, we elected for close surveillance rather than systemic corticosteroids. Recovery was uneventful. Constitutional symptoms resolved within four weeks, and serial blood counts showed steady normalization of both the leukocytosis and the eosinophilia (Table 2). At the 15-month review, the patient remained entirely asymptomatic, with no palpable lymphadenopathy and a normal eosinophil count.

## 3. Literature Review

**Figure 4:**
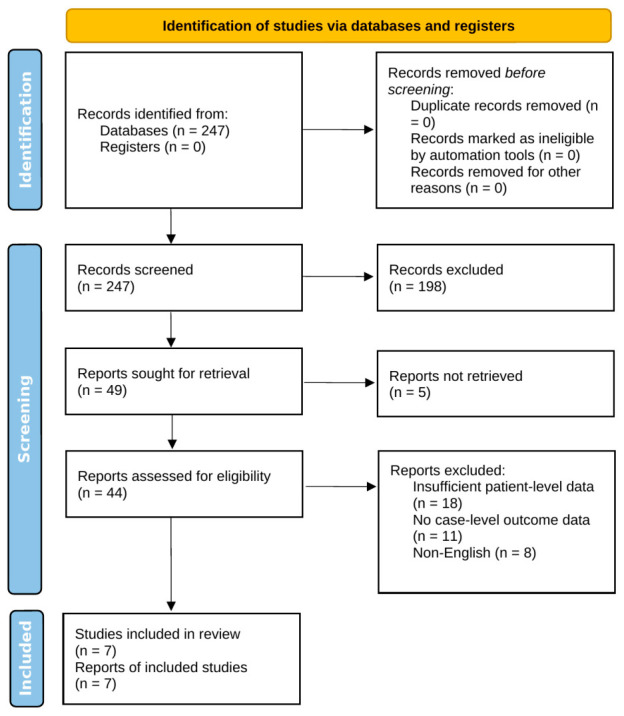
PRISMA 2020 flow diagram summarizing identification, screening, and selection of the studies included in the focused literature review (n = 7; Table 3). The reported numbers are representative of the screening process for this focused review.

To place our findings in context, we performed a focused review of the literature on KD, with particular attention to pediatric and non-Asian patients. PubMed/MEDLINE was searched from database inception to May 2026 using combinations of the terms “Kimura disease,” “eosinophilic lymphogranuloma,” “case report,” “pediatric,” “children,” “Middle East,” “Saudi Arabia,” and “treatment.” The search was restricted to English-language publications. Titles and abstracts were screened against predefined inclusion criteria—histologically confirmed KD reported at the level of the individual patient, with extractable data on age, clinical presentation, treatment and outcome—and the reference lists of relevant articles were hand-searched for additional reports. After exclusion of records that lacked individual patient-level data, seven studies that best illustrate the geographic, age-related and therapeutic spectrum of the disease were selected for detailed comparison with the present case (Table 3). The selection process is summarized in Figure 4, presented in accordance with the PRISMA 2020 statement [16]. This focused approach was intended to contextualize a single case rather than to constitute an exhaustive systematic review.

### 3.1. Geographic Distribution

Although KD is still regarded as an East Asian entity, our review identified well-documented cases across markedly different regions, including the Middle East (Saudi Arabia and Egypt), Africa (Ethiopia), Europe (Portugal) and numerous non-Asian Western series [4,10,11,17,18]. AlGhamdi and colleagues described the only previous Saudi pediatric case in 2016—a 16-year-old boy with cervical lymphadenopathy managed with surgery followed by prolonged corticosteroids [11]. The present case is therefore the second pediatric Saudi report, and it is notable for achieving remission without steroids.

**Table 3:** Representative reports of Kimura disease, with emphasis on pediatric and non-Asian cases, compared with the present case.

Study (Reference)	Country	Patients/Age	Treatment	Outcome/Recurrence (Follow-Up)
AlGhamdi et al. [11]	Saudi Arabia	1; 16 y	Surgery + corticosteroids	Remission; no recurrence reported
Prasad et al. [19]	India	18; pediatric	Surgery vs. steroids	Lower recurrence with surgery (9-year study)
Xu et al. [8]	China	29; pediatric	Mixed modalities	≈60% recurrence irrespective of therapy
Mai et al. [9]	China	11; median 14 y	Surgery ± steroids	Variable; recurrences noted in series
Fouda et al. [17]	Egypt	1; adult	Immunosuppression	Remission; renal involvement at 18 mo
Anbessie [18]	Ethiopia	1; adult	Corticosteroids	Relapse on corticosteroid taper
Zhao et al. [20]	China	Case series	Surgery/steroids	Recurrence common after steroids
Present case	Saudi Arabia	1; 16 y	Surgery alone	No recurrence; 15-mo follow-up

y, years; mo, months.

### 3.2. Pediatric Presentation

Mai and colleagues reported the largest recent pediatric series, comprising 11 Chinese children with a median age of 14 years, in whom the post-auricular region and parotid gland were most often involved [9]. Xu and colleagues collated 29 Chinese pediatric cases and recorded a sobering 60% recurrence rate irrespective of the treatment chosen [8]. In a 9-year prospective Indian study of 18 children, Prasad and colleagues found surgery superior to corticosteroids for preventing recurrence [19].

### 3.3. Treatment and Outcomes

Reported strategies span surgical excision, corticosteroids, radiotherapy and a range of immunosuppressive or steroid-sparing agents (Table 3). Corticosteroids reliably shrink lesions and relieve symptoms, but relapse on tapering is frequent, with rates of roughly 40–80% [15,17]. Anbessie, for example, described an Ethiopian patient who relapsed after steroid withdrawal and ultimately required a very gradual taper over several months [18]. By contrast, complete surgical excision can secure lasting remission in localized disease, with durable control reported in approximately 25–64% of such cases and generally better outcomes in pediatric series [20,21]. Our patient adds to this evidence, illustrating that excision alone may suffice for localized pediatric KD while avoiding the considerable toxicity of long-term corticosteroids in a growing adolescent.

### 3.4. Renal Involvement

Renal complications are an important and sometimes overlooked feature of KD, reported in 10–60% of patients and usually manifesting as proteinuria or frank nephrotic syndrome [22]. Fouda and colleagues documented an Egyptian patient who developed membranous glomerulonephritis 18 months after disease onset and required combination immunosuppression [17]. Our patient showed no renal involvement, and renal function has remained normal throughout follow-up; nonetheless, the association underscores the need for periodic urinalysis during surveillance.

## 4. Discussion

Taken together, this case and the accompanying review carry several practical messages. The first is geographic: KD is not confined to East Asia and should enter the differential diagnosis of any patient with lymphadenopathy and eosinophilia, whatever their ethnicity. The growing number of reports from the Middle East, Africa and Western countries suggests that the disease is under-recognized rather than truly absent in these settings, and that diagnostic delay often reflects unfamiliarity rather than rarity [4,10].

A central challenge in KD is its overlap with several disorders that share lymphadenopathy or eosinophilia, and these were considered carefully in our patient. Classical Hodgkin lymphoma can cause cervical lymphadenopathy with blood eosinophilia, but the absence of Reed–Sternberg cells and the benign reactive architecture on excisional biopsy argued against it. Eosinophilic granulomatosis with polyangiitis (EGPA), a small-vessel vasculitis associated with asthma and eosinophilia, was unlikely given the negative ANCA serology and the lack of pulmonary infiltrates, neuropathy, or other organ involvement. Angiolymphoid hyperplasia with eosinophilia (ALHE) is the principal histological mimic; however, ALHE typically presents as superficial dermal papules and is characterized by plump, epithelioid (“histiocytoid”) endothelial cells, in contrast to the deep nodal involvement, florid germinal-center hyperplasia and eosinophilic microabscesses that characterize KD [3]. Hypereosinophilic syndrome was excluded by the reactive bone marrow and negative clonality studies, which indicated a secondary, reactive eosinophilia rather than a clonal or idiopathic process [4]. Finally, Langerhans cell histiocytosis was excluded immunohistochemically by the negative CD1a and Langerin staining.

From a diagnostic standpoint, tissue remains decisive. Cross-sectional imaging—ultrasonography, computed tomography and magnetic resonance imaging—usually shows lymphadenomegaly with preserved nodal architecture and associated soft-tissue masses, but these appearances are not specific and cannot distinguish KD from other causes of nodal enlargement; imaging is therefore best regarded as complementary to histology rather than diagnostic in its own right. Incisional or excisional biopsy with histopathological examination remains the diagnostic gold standard, as it was in our patient. The marked leukocytosis and eosinophilia may also mislead the clinician toward an acute suppurative process; here, laboratory biomarkers can help, since serum procalcitonin typically remains normal in KD but is elevated in purulent lymphadenitis [23], whereas the delta neutrophil index may rise in both conditions and is therefore less discriminating [24].

**Figure 5:**
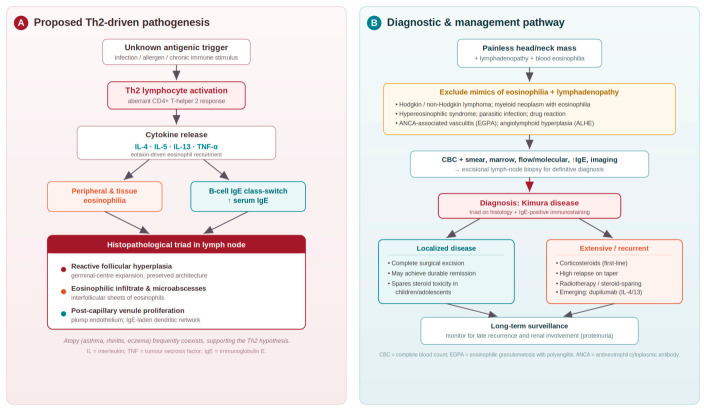
Schematic overview of Kimura disease. (**A**) Proposed T-helper-2 (Th2)-driven pathogenesis, in which an unknown trigger drives Th2 cytokine release (IL-4, IL-5, IL-13, TNF-α), producing eosinophilia and IgE class-switching and, ultimately, the characteristic histopathological triad. (**B**) A practical diagnostic and management pathway, from initial presentation and exclusion of mimics to biopsy-based diagnosis, extent-based treatment and long-term surveillance. Long-term surveillance is intended to incorporate periodic urinalysis and renal monitoring for proteinuria, given the recognized association of KD with renal involvement.

The second message concerns treatment. Our patient supports a stratified approach in which therapy is matched to disease extent and to the individual. Corticosteroids remain a reasonable first choice for extensive or recurrent disease, but the high relapse rate on tapering is a real limitation, and prolonged steroid exposure is especially undesirable in a growing adolescent, in whom growth suppression, metabolic disturbance and reduced bone density are tangible risks. Where disease is localized and can be completely excised, surgery alone may achieve durable remission; published series report lasting control in roughly a quarter to two-thirds of such cases, with the most favorable results in children [19,21]. Figure 5 summarizes the proposed pathogenesis alongside a pragmatic diagnostic and management pathway that integrates these considerations.

Third, the coexisting asthma in our patient fits the proposed Th2-mediated mechanism and the well-described link between KD and atopy. This association is not merely academic: it has prompted interest in targeted biologic therapy, and isolated reports describe benefit from dupilumab, a monoclonal antibody directed against the shared IL-4/IL-13 receptor subunit, in refractory disease [22]. Beyond dupilumab, anecdotal experience points to possible benefit from other agents that target type 2 inflammation, including omalizumab (anti-IgE) and mepolizumab (anti–IL-5), in patients with refractory or recurrent disease [13,15]. It should be emphasized, however, that biologic agents are not first-line therapy for KD; they are reserved for refractory, recurrent, or unresectable disease that has failed conventional measures, and the supporting evidence remains limited to small series and isolated case reports. Reported predictors of recurrence include incomplete resection, multifocal or large lesions, high pretreatment eosinophil counts and very high serum IgE, and corticosteroid-only management with relapse on tapering; these factors help identify patients in whom closer surveillance or escalation to systemic or biologic therapy may be warranted [8,19].

Several limitations deserve mention. Follow-up, while reassuring at 15 months, remains relatively short for a disease that can recur years—even decades—after apparent remission, so continued surveillance is essential. In addition, a steroid-free strategy worked well here because the disease was localized and fully resected; it should not be generalized uncritically to patients with multifocal or unresectable disease, in whom treatment must be individualized. Finally, serial serum IgE concentrations were not measured during follow-up; normalization of the peripheral eosinophil count (Table 2) served as the principal laboratory marker of disease control, and the absence of longitudinal IgE data is acknowledged as a limitation.

## 5. Conclusions

Kimura disease should be considered whenever lymphadenopathy is accompanied by eosinophilia, regardless of the patient’s geographic origin. Diagnosis rests on the histopathological triad of reactive follicular hyperplasia, an eosinophilic infiltrate with microabscesses, and vascular proliferation, supported by IgE-positive immunostaining. Because its presentation overlaps with lymphoma, EGPA, angiolymphoid hyperplasia with eosinophilia, and hypereosinophilic syndrome, histopathological confirmation is essential before treatment, and management should be individualized to disease extent. Although corticosteroids remain the conventional treatment, their high relapse rate on tapering is an important drawback; in localized pediatric disease, complete surgical excision can achieve sustained remission while sparing young patients from steroid toxicity, as illustrated by our patient. Given the potential for late recurrence and renal involvement, long-term follow-up that includes periodic urinalysis is advisable.

## Figures and Tables

**Table 1:** Summary of the diagnostic workup performed to exclude hematological malignancy and other causes of eosinophilia.

Investigation	Result
White-cell count	40 × 10^9^/L (leukocytosis)
Absolute eosinophil count	6.5 × 10^9^/L (36% of leukocytes)
Hemoglobin/platelets	Within normal limits
Peripheral blood smear	Mature eosinophilia; no blasts or dysplasia
Bone-marrow aspirate and trephine	Reactive eosinophilic hyperplasia; no malignancy
Flow cytometry	No clonal B- or T-cell population
*BCR-ABL1*	Not detected
*JAK2* V617F	Not detected
*CALR*/*MPL* mutations	Not detected
*PDGFRB* rearrangement (FISH)	Negative
Serum tryptase	Normal
c-ANCA/p-ANCA	Negative
Serum IgE	Markedly elevated (2480 IU/mL; reference <100 IU/mL)
Stool ova and parasites; Strongyloides and Toxocara serology; HIV serology	Negative
CT neck/chest/abdomen/pelvis	Isolated right cervical lymphadenopathy

ANCA, antineutrophil cytoplasmic antibody; CT, computed tomography; FISH, fluorescence in situ hybridization; IgE, immunoglobulin E.

**Table 2:** Trend of the peripheral blood counts from presentation through follow-up.

Parameter	At Presentation	~6 Weeks Post-Excision	15 Months
White-cell count (×10^9^/L)	40	14	8
Eosinophils (%)	36	12	4
Absolute eosinophils (×10^9^/L)	6.5	1.7	0.3

Values are approximate and illustrate the progressive normalization of the eosinophil count after surgical excision.

## Data Availability

The data supporting the findings of this report are contained within the article. Further de-identified information is available from the corresponding author on reasonable request, subject to patient-privacy constraints.

## Abbreviations

The following abbreviations are used in this manuscript:

AEC	Absolute eosinophil count
ALHE	Angiolymphoid hyperplasia with eosinophilia
ANCA	Antineutrophil cytoplasmic antibody
CBC	Complete blood count
CT	Computed tomography
EGPA	Eosinophilic granulomatosis with polyangiitis
FISH	Fluorescence in situ hybridization
H&E	Hematoxylin and eosin
IgE	Immunoglobulin E
IL	Interleukin
KD	Kimura disease
LCH	Langerhans cell histiocytosis
TNF-α	Tumor necrosis factor-alpha

## References

[1] Kimm H.T., Szeto C. (1937). Eosinophilic hyperplastic lymphogranuloma, comparison with Mikulicz’s disease. Chin. Med. J..

[2] Kimura T., Yoshimura S., Ishikawa E. (1948). On the unusual granulation combined with hyperplastic changes of lymphatic tissues. Trans. Soc. Pathol. Jpn..

[3] Kuo T.T., Shih L.Y., Chan H.L. (1988). Kimura’s disease. Involvement of regional lymph nodes and distinction from angiolymphoid hyperplasia with eosinophilia. Am. J. Surg. Pathol..

[4] Chen H., Thompson L.D., Aguilera N.S., Abbondanzo S.L. (2004). Kimura disease: A clinicopathologic study of 21 cases. Am. J. Surg. Pathol..

[5] Wang D.Y., Mao J.H., Zhang Y., Gu W.Z., Zhao S.A., Chen Y.F., Liu A.M. (2009). Kimura disease: A case report and review of the Chinese literature. Nephron Clin. Pract..

[6] Ye P., Wei T., Yu G.Y., Wu L.L., Peng X. (2016). Comparison of local recurrence rate of three treatment modalities for Kimura disease. J. Craniofac. Surg..

[7] Zhang Y., Bao H., Zhang X., Yang F., Liu Y., Li H., Lu J. (2021). Kimura’s disease: Clinical characteristics, management and outcome of 20 cases from China. Clin. Exp. Rheumatol..

[8] Xu X., Fu J., Fang Y., Liang L. (2011). Kimura disease in children: A case report and a summary of the literature in Chinese. J. Pediatr. Hematol. Oncol..

[9] Mai Y., Wang Y., Sun P., Jing Z., Dong P., Liu J. (2023). Kimura disease in children: A report of 11 cases and review of the literature. Front. Pediatr..

[10] Abuel-Haija M., Hurford M.T. (2007). Kimura disease. Arch. Pathol. Lab. Med..

[11] AlGhamdi S.A., Al-Khatib T., Marzouki H.Z., AlGarni M.A. (2016). Kimura disease: A case report in a Saudi child. Saudi Med. J..

[12] Hosoki K., Hirayama M., Kephart G.M., Kita H., Nagao M., Uchizono H., Toyoda H., Senba Y., Imai Y., Komada Y. (2012). Elevated numbers of cells producing interleukin-5 and interleukin-10 in a boy with Kimura disease. Int. Arch. Allergy Immunol..

[13] Kim W.J., Kim H.K. (2022). Current concepts of Kimura disease: Pathophysiology and evolution of treatment. Arch. Craniofac. Surg..

[14] Sun Q.F., Xu D.Z., Pan S.H., Ding J.G., Xue Z.Q., Miao C.S., Cao G.J., Jin D.J. (2008). Kimura disease: Review of the literature. Intern. Med. J..

[15] Lee C.C., Feng I.J., Chen Y.T., Weng S.F., Chan L.P., Lai C.S., Lin S.D., Kuo Y.R. (2022). Treatment algorithm for Kimura’s disease: A systematic review and meta-analysis of treatment modalities and prognostic predictors. Int. J. Surg..

[16] Page M.J., McKenzie J.E., Bossuyt P.M., Boutron I., Hoffmann T.C., Mulrow C.D., Shamseer L., Tetzlaff J.M., Akl E.A., Brennan S.E. (2021). The PRISMA 2020 statement: An updated guideline for reporting systematic reviews. BMJ.

[17] Fouda M.A., Gheith O., Refaie A., El-Saeed M., Bakr A., Wafa E., Abdelraheem M., Sobh M. (2011). Kimura disease: A case report and review of the literature with a new management protocol. Int. J. Nephrol..

[18] Anbessie Z.M. (2024). Kimura’s disease: A case report. J. Med. Case Rep..

[19] Prasad B.K., Deviprasad D., Rao I.S., Rao D.B. (2007). Kimura’s disease in children: A 9 years prospective study. Int. J. Pediatr. Otorhinolaryngol..

[20] Zhao F., Zhou M., Mao A., Zhang Y., Chen Y. (2024). Kimura disease: A detailed analysis of clinical and radiological manifestations in a retrospective case series. J. Inflamm. Res..

[21] Harshi D., Nagpal R., Baliyan A., Alva S.R. (2019). Kimura disease: Case report and brief review of literature. Med. Pharm. Rep..

[22] Brito M.T., Baptista D., Pereira E., Fonseca E., Almeida J.S., Almeida J.S. (2023). Kimura’s disease: A literature review based on a clinical case. Cureus.

[23] Yankov Y.G., Bocheva Y.D. (2023). Comparative characterization of procalcitonin (sensitivity, specificity, predictability, and cut-off reference values) as a marker of inflammation in odontogenic abscesses of the head and neck in the female population. Cureus.

[24] Yankov Y.G. (2023). Delta neutrophil index as a new marker of purulent inflammation in men with non-odontogenic abscesses of the neck. Cureus.

